# Differentiation of Pluripotent Stem Cells for Disease Modeling: Learning from Heart Development

**DOI:** 10.3390/ph17030337

**Published:** 2024-03-05

**Authors:** Congwu Chi, Truman J. Roland, Kunhua Song

**Affiliations:** 1 Heart Institute, University of South Florida, Tampa, FL 33602, USA; congwuchi@usf.edu (C.C.); trumanroland@usf.edu (T.J.R.); 2 Department of Internal Medicine, University of South Florida, Tampa, FL 33602, USA; 3 Center for Regenerative Medicine, University of South Florida, Tampa, FL 33602, USA

**Keywords:** heart development, signaling pathways, pluripotent stem cells, cardiac differentiation, disease modeling

## Abstract

Heart disease is a pressing public health problem and the leading cause of death worldwide. The heart is the first organ to gain function during embryogenesis in mammals. Heart development involves cell determination, expansion, migration, and crosstalk, which are orchestrated by numerous signaling pathways, such as the Wnt, TGF-β, IGF, and Retinoic acid signaling pathways. Human-induced pluripotent stem cell-based platforms are emerging as promising approaches for modeling heart disease in vitro. Understanding the signaling pathways that are essential for cardiac development has shed light on the molecular mechanisms of congenital heart defects and postnatal heart diseases, significantly advancing stem cell-based platforms to model heart diseases. This review summarizes signaling pathways that are crucial for heart development and discusses how these findings improve the strategies for modeling human heart disease in vitro.

## 1. Introduction

Heart disease is the leading cause of death worldwide. According to the Centers for Disease Control and Prevention, about 610,000 Americans annually die of heart disease—one in every four deaths. Heart disease costs about $200 billion annually in the United States. Common conditions in heart disease include congenital heart defects, arrhythmia (irregular heartbeats), blood vessel disorders, heart valve defects, cardiomyopathy, and heart failure. All these conditions worsen patients’ heart function and life quality. Additionally, heart disease can be triggered and exacerbated by many lifestyle-related behaviors and pathological conditions, such as tobacco use, environmental factors, nutrition, obesity, high blood pressure, excess cholesterol, metabolic syndromes, kidney disease, and pregnancy [1]. Despite the heavy social burden of heart disease, treatment options are minimal. The heart is the first functional organ that emerges during embryogenesis due to its vital role in fetal oxygen and nutrient distribution [2,3]. From the evolutionary point of view, the heart’s morphology evolved from a relatively simple, single tube-like structure in insects, to comprising four chambers in mammals. Heart development involves tightly regulated spatial and temporal interactions among cell types and crosstalk between signaling pathways. This process requires the sequential activation of gene regulation networks and the spatial organization of diverse cell populations, eventually forming the three-dimensional structure of the heart [4,5]. Any disruption of these steps can contribute to heart malformation and cardiac dysfunction. Improving our understanding of heart development mechanisms significantly advances in vitro disease models, such as human-induced pluripotent stem cell-based platforms. This review summarizes mechanisms underlying heart development and their applications in developing in vitro models for disease modeling and drug discovery.

## 2. Cardiac Morphogenesis

### 2.1. Heart Types

The circulation of fluid carrying oxygen, nutrients, and signals is a critical function for a great number of multicellular organisms, and is accomplished through varied but homologous structures. Most invertebrates and arthropods use open circulatory systems where a tube-like heart pumps hemolymph (respiratory proteins equivalent to hemoglobin) throughout the body [6]. Vertebrates and some mollusks use closed circulatory systems, with the heart divided into multiple chambers. The main benefits of closed circulatory systems are a faster blood flow and better-defined directionality than open systems. In fish, two-chambered hearts draw deoxygenated blood into a single atrium and then pump it out through a single ventricle. After leaving the heart, the deoxygenated blood is oxygenated by the gills before circulating the body [7]. Valve-controlled chamber systems are used in hearts with three (two atria and one ventricle in reptiles/amphibians) or four chambers (two atria and two ventricles in mammals). These animals use a pulmonary circuit, which oxygenates blood in the lungs after it enters and before it exits the heart [8]. In three-chambered hearts, deoxygenated and oxygenated blood mix in a single ventricle. Four-chambered hearts separate deoxygenated and oxygenated blood into two ventricles, which more efficiently uses oxygen.

### 2.2. Heart Development

As a critical organ, the heart develops and forms its shape during early embryogenesis. During gastrulation, the blastula folds in on itself to form a crescent structure. Cells in the blastula rearrange themselves and migrate to create the three germ layers: endoderm (inner layer), mesoderm (middle layer), and ectoderm (outer layer) [9]. Organs develop from the germ layers through cell determination and differentiation. For example, most heart tissue arises from the mesoderm [10]. Different genes are turned on or off during heart development, determining the cells’ fate, commitment, and differentiation. At mouse embryonic day 7.5 (E7.5), equivalent to 2 weeks after human gestation, a pool of cardiac progenitors organizes into a crescent structure known as the first heart field (FHF) [5,11]. FHF cells are considered the earliest population of cardiac progenitors in the embryonic heart [11].

**Figure 1:**
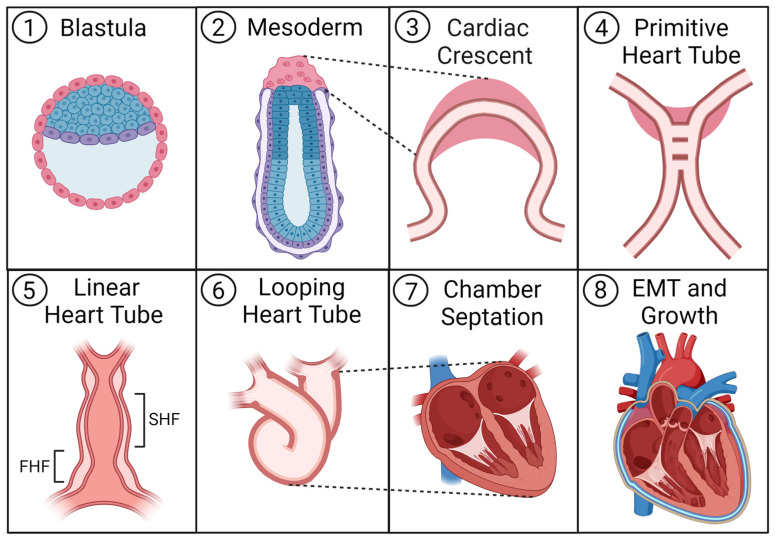
Heart development: (1) The embryonic blastula provides stem tissue which differentiates to ectoderm, mesoderm, or endoderm. (2) Mesodermal tissue appears in the ALPM of the implanted embryo, giving rise to cardiac mesoderm progenitors. (3) These cardiac progenitors facilitate the development of primitive endothelial tubes in close proximity to one another. (4) The two sides of the endothelial tubing begin to fuse together and establish a new vessel known as the primitive heart tube. (5) Critical cardiac progenitor populations known as the FHF and SHF emerge as distinct regions within the linear heart tube. Around this time, the heart begins beating. (6) Endogenous signaling pathways incite the heart tube to first loop, then fold into new structures. (7) After the heart tube folds, tissue remodeling produces the septum of the heart, as well as, eventually, all four chambers. (8) As cardiomyocyte populations are established, they expand and eventually begin to undergo physiological hypertrophy and growth to prepare for the contractile demands of adult life. Additionally, the tissue is diversified with new cell types as the epicardium begins to spread around the heart and undergo EMT (alongside parts of the endocardium) to produce further fibroblasts, vascular smooth muscle cells, pericytes, and some endothelial cells, which support cardiac function. ALPM, anterior lateral plate mesoderm; EMT, epithelial-to-mesenchymal transition; FHF, first heart field; SHF, second heart field.

FHF formation and development are initiated by mesodermal progenitor cells marked by the expression of a basic helix-loop-helix (bHLH) transcription factor gene, *mesoderm posterior-1* (*Mesp-1*). MESP-1 upregulates gene networks that promote the initialization of mesodermal gene expression and the suppression of endodermal gene expression [12,13]. This allows specific mesodermal progenitors to differentiate between precardiac mesodermal progenitors. Precardiac mesodermal progenitors then migrate and reach the anterior lateral plate mesoderm (ALPM), where they become cardiac mesoderm cells [14,15,16]. The FHF cardiac gene expression program is regulated by several key transcription factors. The expression of transcription factors Ying Yang 1 (YY1) and GATA Binding Protein 4 (Gata4) activates several cardiac genes, including the homeobox transcription factor *Nkx2.5* and T-box transcription factor *Tbx5*. Nkx2.5 and Tbx5 work together to induce cardiac gene expression, such as *atrial natriuretic factor* (*ANF*) and *connexin 40* (*cx40*), and promote the development of the left ventricle [17,18]. Around E8.0, FHF progenitors begin folding towards the embryo midline, forming a linear tube-like structure called the heart tube [19,20,21]. Like those in the FHF, second heart field (SHF) progenitors also arise from mesodermal progenitors and share many similarities with FHF development, except they appear later. The transcription factors ISL LIM Homeobox 1 (Isl1) and Gata4 drive SHF progenitor proliferation [22] and activate expression of the transcription factor gene *heart and neural crest derivatives expressed 2* (*Hand2*), which is required for SHF progenitor expansion [23,24]. SHF differentiation produces many cell types in the right ventricle and atria, including cardiomyocytes (CMs), smooth muscle cells, and endothelial cells [22,25]. FHF and SHF differentiation and migration drive heart tube elongation and rightward bending into the looped heart tube, eventually forming the four-chambered heart [20,26]. Heart development is illustrated in Figure 1.

After the establishment of the early heart populations in the myocardium, endocardium, and epicardium, epithelial-/endothelial-to-mesenchymal (EMT) transition plays an important role in further producing cardiac cell types and architecture. The tightly adjoined mosaic cells of the heart’s epicardium and endocardium undergo signaling which initiates a transition to a plastic mesenchymal phenotype, becoming multiple other cell types. The epithelial cells of the epicardium produce cardiac fibroblasts, mural cells (vascular smooth muscle and pericytes), and some endothelial cells [27]. The endothelial cells of the endocardium can undergo a more transient transition to a mesenchymal phenotype to enable migration and the formation of new structures like the valves of the heart [28], endocardial cushioning, and coronary vasculature [29]. Epithelial cells undergoing EMT also cooperatively contribute mesenchymal cells to these structures as well. While EMT in the endocardium contributes to the formation of the heart’s internal architecture, dysregulated EMT later in life can contribute to valvular and vascular disease [29]. EMT is regulated by several signaling pathways including TGF-β/BMP, VEGF, FGF, PDGF, and Wnt/β-catenin, which also play vital roles in other aspects of cardiogenesis [27,29]. Inflammatory stimulation by TNF, IFN, IL-1B, proteases, and hypoxia can also contribute to EMT [28,29]. Understanding the diverse and multi-faceted roles of signaling pathways in cardiogenesis is key to both our understanding of in vivo systems and our ability to capture these systems in vitro for study and therapy. 

## 3. Signaling Pathways in Heart Development

**Figure 2:**
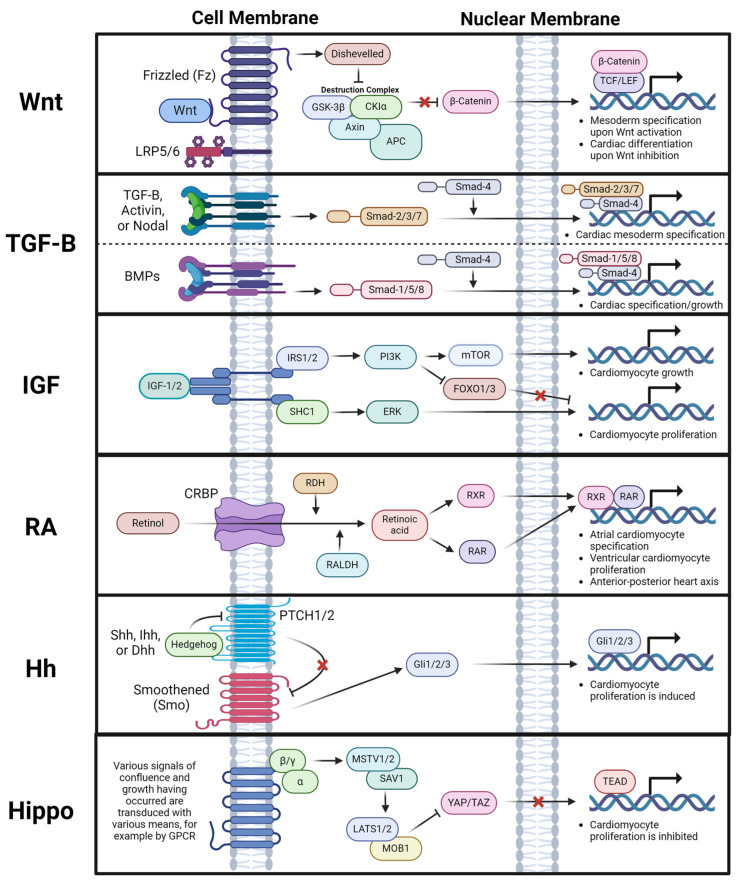
Signaling pathway molecular mechanisms: Several signaling pathways play a major role in tissue development and thus in PSC differentiation, specifically into cardiac tissue. The molecular mechanisms of these pathways are shown as the signal is transduced from outside of the cell, past the cell membrane, through the cytosol, past the nuclear membrane, and into the nucleus where transcription factors mediate changes in gene expression for the given pathway. Wnt signaling uses Wnt proteins as ligands to the Frizzled (Fz) and LRP5/6 co-receptor, where receptor activation recruits Disheveled to arrest the destruction complex, enabling β-catenin accumulation in the cytosol, which triggers its translocation to the nucleus, where mesodermal genes are activated. TGF-Β and BMP signaling occur through their respective receptors mediated by phosphorylated Smad proteins to promote cardiac and cardiac mesoderm genes. IGF signaling occurs when IGF-1/2 activates IGF1R, leading to cardiomyocyte growth through mTOR signaling and cardiomyocyte proliferation through MAPK-ERK signaling. Retinoic acid (RA) occurs when CRBP imports retinol into the cell which is converted to RA by enzymes RDH and RALDH. RA then initiates the translocation of RXR and RAR to the nucleus, which specifies the cardiomyocyte phenotype and the heart’s anterior–posterior axis. Hedgehog (Hh) signaling occurs when a Hh protein variant activates Smoothened (Smo) by inhibiting its inhibitor PTCH1/2. This allows Smo to activate Gli1/2/3, which are translocated to the nucleus to induce cardiomyocyte proliferation. Hippo and Yap/TAZ signaling is mediated by a variety of signals of tissue growth which, when sufficiently present, activate the MSTV1/2 to LATS1/2 cascade to inhibit the proliferative YAP/TAZ pathway and to induce arrest of cardiomyocyte proliferation. APC, adenomatous polyposis coli; BMP, bone morphogenic protein; CK1α, casein kinase one alpha; CRBP, cell retinol binding protein; FOXO1/3, Forkhead box protein O1/3; Gli1/2/3, glioma-associated oncogene family zinc finger one/two/three; GPCR, G-protein coupled receptor; GSK3β, Glycogen synthase kinase-3 beta; Hh (Shh/Ihh/Dhh), (sonic/Indian/desert) hedgehog; IGF-1/2, insulin-like growth factor one/two; IRS1/2, insulin receptor substrate ine/two; LATS1/2, large tumor suppressor kinase one/two; LPR5/6, low-density lipoprotein receptor-related protein five/six; mTOR, the mammalian target of rapamycin (mTOR); MAPK-ERK, mitogen-activated protein kinase (MAPK)-extracellular signal-regulated kinase (ERK); MOB1, monopolar spindle-one-binder protein one; MST1/2, macrophage stimulating protein one/two; PI3K, phosphatidyl-inositol-3 kinase; PSC, pluripotent stem cell; PTCH1/2, protein patched homolog one/two; RA, Retinoic acid; RAR, Retinoic acid receptor; RALDH, retinaldehyde dehydrogenases; RDH, retinoid dehydrogenase; RXR, Retinoic acid X receptor; SAV1, tumor suppressor Salvador homolog one; SHC1, Src homology and collagen-transforming protein one; Smad, small (worm phenotype) mothers against decapentaplegic; Smo, smoothened; TEAD, Transcriptional enhancer factor/TEA domain family; TCF/LEF, T-cell factor/lymphoid enhancer factor family; TGF-β, transforming growth factor beta; Wnt, wingless-related integration site; YAP/TAZ, yes-associated protein/transcriptional coactivator with PDZ-binding motif.

Organogenesis involves the spatial and temporal assembly of multiple cell types. Cellular proliferation, migration, and differentiation must be precisely regulated by cell signaling [30]. Therefore, understanding signaling pathways which control heart development shows how cardiac progenitor cells appear and interact, and elucidates the basis of congenital cardiac malformations. Several methods of signal transmission must also be considered, especially in the context of crosstalk between cell types and the circulation of signals to and from the whole-body. Many cell signals act over short distances, such as the cell–cell or cell–ECM interactions involved in mechanotransduction and juxtacrine cues. Secreted factors and nucleic acids may also act as paracrine cues within and between regions of tissue, while hormones can act through bodily circulation in an endocrine manner. The role of extracellular vesicles (EVs) including exosomes has become increasingly recognized as an important mode of signal transmission between cell populations [31,32]. Thus, many efforts have been made to unravel the signaling pathways underlying heart development. Here, we highlight some of signaling pathways that are essential for heart development. The molecular mechanisms of these signaling pathways are illustrated in Figure 2.

### 3.1. Wnt Signaling

The Wnt gene was first identified as a gene required for embryogenesis in fruit flies in the 1980s in a study on developmental mutations [33]. Mutations in *Wnt* cause the loss of wing tissue during embryonic development, hence the original gene name, *Wingless*. *Wnt* genes are highly conserved with homologs across species [34,35,36]. Wnt genes represent a large family of genes. In humans, there are 19 genes encoding Wnt proteins, including Wnt1, Wnt2, Wnt2b (Wnt13), Wnt3, Wnt3a, Wnt4, Wnt5a, Wnt5b, Wnt6, Wnt7a, Wnt7b, Wnt8a, Wnt8b, Wnt9a (Wnt14), Wnt9b (Wnt14b), Wnt10a, Wnt10b, Wnt11, and Wnt16 [37]. Two types of signaling pathways are driven by Wnt proteins: the non-canonical Wnt signaling pathway and the β-catenin-medicated canonical Wnt pathway. Based on their functional characteristics, Wnt proteins can be divided into two categories: Wnt1, Wnt2, Wnt2b, Wnt3, Wnt3a, Wnt7a, Wnt8, Wnt8b, and Wnt10a, which are involved in the canonical signaling pathway, and Wnt4, Wnt5a, and Wnt11, which activate the non-canonical signaling pathway [38]. However, Wnt proteins have varying degrees of selectivity towards these two pathways and can activate either of the two pathways depending on their spatiotemporal availability [39].

In canonical Wnt signaling, signal transduction starts with a Wnt ligand binding to its receptor Frizzled (Fz) and co-receptor lipoprotein, receptor-related proteins 5 and 6 (LRP5/6). The binding recruits the scaffold protein Disheveled (Dvl) and causes the dissociation of the “destruction complex”, which is composed of casein kinase 1 (CK1), glycogen synthase kinase-3β (GSK-3β), and the scaffolding proteins adenomatous polyposis coli (APC), Axin1, and Axin2. Without an upstream signal, the “destruction complex” phosphorylates the amino-terminal region of β-catenin, resulting in recognition by β-Trcp, an E3 ubiquitin ligase subunit, and the subsequent ubiquitination and proteasomal degradation of β-catenin. However, the dissociation of the “destruction complex” inhibits the phosphorylation of β-catenin and thereby stabilizes it. The cytoplasmic accumulation of β-catenin drives its translocation into the nucleus to associate with TCF/LEF DNA consensus sequence binding partners, which activates the expression of Wnt target genes (Figure 2) [40]. By contrast, non-canonical Wnt signaling refers to all Wnt-dependent signaling pathways which do not lead to the nuclear translocation of stabilized β-catenin [41]. 

The roles of the Wnt signaling pathways in heart development are complicated, mainly due to the large number of Wnt proteins with both canonical and non-canonical pathways. Wnt signaling can both promote or inhibit cardiogenesis. Studies in amphibians, fish, and mice have revealed the roles of canonical Wnt signaling (Wnt/β-catenin signaling) during cardiogenesis. In frog embryos, the activation of Wnt/β-catenin signaling in the early embryonic stage is required to establish the anterior–dorsal axis, which specifies the site for mesoderm formation [42,43]. Embryos depleted of maternal β-catenin fail to form the anterior–dorsal axis and mesoderm. On the other hand, the overexpression of β-catenin in both frogs and zebrafish causes the formation of secondary axes and ectopic expression of mesoderm marker genes [43,44,45]. Wnt/β-catenin signaling activates mesodermal gene expression through the FGF3-MAPK pathway [46]. Wnt/β-catenin signaling has a similar role in mice. Homozygous β-catenin in knockout mice prevents mesoderm formation [47]. Using tissue-specific in vivo genetic manipulation in mice, Kwon et al. found that β-catenin is required for cardiac progenitor development and proliferation [48]. Canonical Wnt pathway suppression contributes to cardiogenesis defects in Down syndrome [49]. These studies demonstrate that canonical Wnt signaling is essential for mesoderm formation and cardiac progenitor commitment during early heart development. In contrast, Wnt/β-catenin signaling switches to an inhibitory role in chicken and frog hearts after mesoderm formation. Nkx2.5 expression was inhibited when Wnt-3a was ectopically overexpressed in the precardiac mesoderm. This finding indicates that ectopic Wnt/β-catenin signals suppress heart formation [50]. Furthermore, the Wnt inhibitors Dkk-1 and Crescent, in chicken and frog embryos, promote heart-specific gene expression in the cardiac mesoderm [50,51]. In the noncardiogenic ventral marginal zone (VMZ) mesoderm, the ectopic expression of GSK-3β, which enables the degradation of β-catenin, induces heart-specific genes’ expression [51]. In β-catenin knockout mice, the endoderm induces ectopic heart formation in parallel with normal heart formation in the mesoderm, indicating that canonical Wnt signaling is required to suppress cardiac differentiation in the endoderm. Suppression of Wnt signaling is essential for differentiating cardiac progenitors into CMs during the later stages of cardiogenesis [52]. Together, these findings suggest a multiphasic role of canonical Wnt signaling in heart development. Wnt/β-catenin signaling promotes cardiac progenitor commitment and expansion during early heart development. In contrast, inhibition of this pathway is essential for later heart development. This conclusion is supported by studies in zebrafish and mouse embryonic stem cells (ES cells) [53,54]. For example, canonical Wnt/β-catenin signaling regulates heart development temporally and bi-phasically [54]. The activation of Wnt/β-catenin signaling induces cardiac specification at early embryogenesis. Inhibition of the Wnt/β-catenin pathway in the later heart development stage is critical for cardiac differentiation and maturation; therefore, it is important for heart regeneration and injury repair.

Non-canonical Wnt signaling also influences heart development. The most studied non-canonical Wnt signaling pathways are the Wnt/Ca^2+^ pathway and Wnt/planar cell polarity (PCP) pathway [55]. The Wnt/Ca^2+^ pathway activates Calcium/calmodulin-dependent protein kinase II (CaMKII) and the nuclear factor of activated T cells (NFAT) via increasing the intracellular Ca^2+^ concentration [55]. In zebrafish, Wnt/Ca^2+^ signaling is required for establishing an electrical gradient in the plane of the developing cardiac epithelium through modulation of the ion channel function, which is necessary for proper heart development [56]. The Wnt/PCP pathway induces the phosphorylation of c-Jun N-terminal kinase (JNK) [55]. Several lines of evidence have shown that the Wnt/JNK-mediated pathway promotes cardiac specification in the early mesoderm [57,58,59]. In a later development stage, the Wnt/JNK-mediated non-canonical pathway is critical for terminal CM differentiation [60,61]. However, a study showed that Wnt/JNK activation is not required for cardiac specification [61]. These studies indicate that the roles of the Wnt/JNK-mediated non-canonical pathway in heart development need to be further investigated. 

### 3.2. TGF-β Signaling

The transforming growth factor β (TGF-β) superfamily is a large and continuously expanding group of structurally related growth factors, including TGF-β, nodal growth differentiation factor (Nodal), Activin, bone morphogenetic proteins (BMPs), growth and differentiation factor (GDF), etc [62]. Since the TGF-β superfamily was first discovered in cancer cells in the 1970s [63], these growth factors have been shown to play essential roles in a wide range of biological processes during embryonic development and adult tissue homeostasis [64,65]. In endoplasmic reticulum, the growth factors in the TGF-β superfamily are synthesized as inactive proprotein forms which contain the C-terminal prodomain, also known as latency associated peptide (LAP). In the Golgi apparatus, LAP is cleaved by the Furin family of proprotein convertases, but this alone does not release mature growth factors. LAP and mature growth factor form a latent complex through noncovalent bonding which maintains growth factors in inactive forms before their secretion from the cell. After secretion, LAP binds integrins with the help of other extracellular matrix (ECM) proteins (i.e., fibylin-1 and fibronectin), changing the LAP configuration to release mature growth factors [62,66,67]. The receptors for all TGF-β superfamily ligands are transmembrane. They share similar structures, including a ligand-binding extracellular domain (N-terminal), a transmembrane domain, and an intracellular domain (C-terminal) with serine/threonine protein kinase activity. Some ligands only use specific receptors for signaling; for example, TGF-β only binds to TGF-β receptors. However, these ligands often share receptors, for instance, Nodal and Activin [66]. TGF-β signaling functions through Smad-mediated canonical or Smad-independent non-canonical pathways [68]. The binding of the extracellular ligand activates the receptor’s kinase activity, phosphorylating Smad-type second messengers to activate the transcription of target genes. TGF-β/Nodal/Activin exerts biological effects through the Smad-2, Smad-3, Smad-4, and Smad-7, whereas BMPs and GDF signal through the Smad-1, Smad-5, and Smad-8 [66]. Phosphorylated Smad proteins bind to distinct genome loci with the help of sequence-specific transcription factors, often expressed in a cell type-dependent manner (Figure 2). For example, studies in frogs have found that the evolutionarily conserved forkhead transcription factor Foxh1 can recruit activated Smad-2/4 complex to Activin response elements (ARE) within the promoters of genes such as *Mix.2* [69,70]. Similar binding sites for Foxh1–Smad complexes have also been found in many mouse genes during embryogenesis and in NODAL-responsive genes in human and fish [71,72,73]. By binding to DNA, Smad proteins act as transcriptional regulators to activate or repress transcription by recruiting different epigenetic modifiers, which further modulate the accessibility of the surrounding chromatin for transcription [66]. 

TGF-β signaling pathways play essential roles during heart development by contributing to the formation of the embryonic axis and cardiac mesoderm. TGF-β signaling is also required for cell proliferation and migration in the developing heart. During early embryonic development, Nodal/Activin-Smad-2/3 signaling is necessary for mesoderm and endoderm specifications. The depletion of Nodal/Activin-Smad-2/3 signaling in Zebrafish embryos disrupts the formation of the dorsal mesoderm, including the heart [74]. Mice carrying a null allele of Nodal cannot gastrulate due to early developmental arrest, while the hypomorphic allele of Nodal causes developmental defects, including abnormal anteroposterior axis positioning, a disorganized mesoderm, and congenital heart defects [75,76]. Studies using mouse ES cells indicate that Nodal/Activin-Smad-2/3 signaling is required for the differentiation of cardiac lineage cells [77,78]. BMP, a subgroup of the TGF-β superfamily, is also essential for heart development. Studies in frogs and mice demonstrate that BMPs are not required for the initial specification of cardiac tissue but are required to maintain cardiac gene expression [16,79,80]. However, BMP signaling is essential for the entire process of mouse heart development, with BMP2 knockout mice dying after heart tube formation [81]. Similarly, BMP4 deletion kills mice during gastrulation [82]. Additionally, the blocking of Smad-independent BMP pathway (TAB1 or TAK1) activity reduces the cardiac progenitor pool size and the number of atrial CMs [83,84]. This evidence supports BMPs as regulators of heart development through Smad-dependent and independent pathways [85].

### 3.3. IGF Signaling

The Insulin-like Growth Factors (IGFs) are a family of evolutionarily conserved ligands, including IGF-1, IGF-2, and insulin [86]. IGF signaling can regulate multiple cellular processes, including proliferation, differentiation, metabolism, and glucose homeostasis [87]. IGFs bind to three types of receptors that have tyrosine kinase activity: the IGF-1 receptor (IGF1R), IGF-2 receptor (IGF2R), and insulin receptor (INSR). When activated, IGF receptors bind to intracellular adaptor proteins such as insulin receptor substrates 1 and 2 (IRS1, IRS2), SHC1, GAB, and CRK. These adaptor proteins are necessary for IGF receptors to activate numerous downstream pathways, including the phosphatidyl-inositol-3 kinase (PI3K)-AKT pathway, the mammalian target of rapamycin (mTOR) pathway, and the mitogen-activated protein kinase (MAPK)-extracellular signal-regulated kinase (ERK) pathway [88].

IGF signaling is essential for CM proliferation and heart growth during heart development. Many different tissues can produce IGFs, but circulating IGF-1 is mainly produced by the liver under the regulation of growth hormones. Other tissues can also produce IGF-1 in an autocrine/paracrine manner [89], while CMs express both IGF-1 and IGF-2 [90]. Studies in chick and zebrafish embryos support IGF-1’s anti-oxidative stress and anti-apoptosis roles in developing cardiac tissues [91,92]. Knockdown of IGF-1 in the embryonic heart of chicks significantly decreased the antioxidant capacity and increased reactive oxygen species (ROS) activity. In tbx5-deficient zebrafish embryos, exogenous recombinant human IGF-I administration ameliorated dysmorphogenesis by suppressed cell apoptosis. The deletion of Igf1 in mice causes stunted growth and perinatal lethality due to severe muscle dystrophy in multiple organs including the heart [93,94]. However, IGF-1’s role during heart development needs further clarification. In contrast, the functions of IGF-2 during heart development have been characterized well. IGF-2 is the main CM mitogen in embryogenesis [95,96]. IGF2 promotes CM proliferation during heart development by activating the ERK-MAPK and PI3K-AKT pathways [95,96,97]. Activation of the ERK-MAPK pathway positively regulates CM proliferation. On the other hand, activation of the PI3K-AKT pathway inhibits the activity of FOXO factors. FOXO1 and FOXO3 are negative regulators of CM proliferation (Figure 2) [98]. IGF-2 signaling can be mediated by two receptors, IGF1R and INSR, sharing 70% of their structure homology [99]. IGF1R and INSR are the primary receptors which mediate IGF signaling in the heart. Global knockout of the *Igf2* ligand gene, or conditional double knockout of both the *Igf1r* and *Insr* genes in the embryonic myocardium by the Cre driven by *Nkx2.5* promoter, each cause a significant decrease in ventricular CM proliferation, causing ventricular wall hypoplasia [95,100]. Consistent with these findings, IGF-2 signaling is required for CM proliferation and heart regeneration in zebrafish [101].

### 3.4. Retinoic Acid Signaling

Retinoic acid (RA) is an active vitamin A metabolite that acts as an essential, diffusible morphogen during embryogenesis through autocrine or paracrine signaling [102]. Vitamin A/retinol is recruited to target cells by plasma retinol-binding protein (PRBP) taken up through Stimulated by Retinoic Acid (STRA) receptors on the cell membrane [103]. Retinol forms a complex in the cell with retinol-binding proteins (CRBP), converting it to retinaldehyde by retinoid dehydrogenases (RDH). Retinaldehyde can be further oxidated by retinaldehyde dehydrogenases (RALDH) to produce RA. To maintain a proper level of cellular RA, retinaldehyde can be converted back to retinol by retinaldehyde reductase (DHRS) for storage and RA can be degraded by cytochrome P450 hydroxylase (CYP26). Cellular RA is translocated to the nucleus to regulate the transcription of target genes with the help of the steroid hormone superfamily of transcription factors, the Retinoic acid receptors (RARs), and the Retinoic acid X receptors (RXRs). Target genes’ regulatory regions contain a signature RA-response element (RARE) (Figure 2) [102].

Maintaining a proper level of RA within the cell is critical for embryogenesis. Excess or deficient RA signaling causes defects in developing organs including the heart [104]. RA signaling plays essential roles in multiple stages of heart development, including the formation of anterior–posterior boundaries of the cardiac mesoderm, CM subtype specification, epicardium and outflow tract development, ventricular wall growth, and coronary arteriogenesis [105]. In the 1950s, multi-organ abnormalities, including those of the heart, were observed in vitamin A-deficient rat embryos [106]. RALDH2 enzyme catalyzes the oxidative step in RA biosynthesis, and its loss of function creates a severe embryonic RA deficiency. Raldh2 knockout mice fail to undergo heart looping and have impaired atrial and sinus venous development [107]. Raldh2 knockout embryos ectopically express anterior SHF markers, such as TBX1, FGF8, and ISL-1, in the posterior region, indicating that a proper RA level is required to restrict the SHF posteriorly. RA signaling is critical for atrial CM specification from human or mouse ES cells. Embryoid bodies (EB) generated from mouse ES cells were treated with RA in early (day 1–5) and late (day 6–10) differentiation days, and their lineage-specific cardiac markers and ion channels were examined. The authors found that RA treatment in early differentiation induced significant atrial CMs sublineage specification [108]. Similarly, RA signaling is also required for atrial CM specification during human ES cell differentiation [109,110], while RA signaling inhibits ventricular myocyte specification [107,111]. However, RA signaling is essential for ventricular development and maturation in the later stages of heart development. RA acts as a mitogen and morphogen during ventricular growth, which involves trabeculation and subsequent thickening of the compact layer (Figure 2). In Raldh2 knockout mouse embryos, abnormal looping results in a single ventricle lacking trabeculae [112,113]. In line with this, ventricular hypoplasia was also observed in vitamin A-deficient rat embryos [106]. 

### 3.5. Hedgehog Signaling

The hedgehog (Hh) signaling pathway can regulate many cell behaviors during embrogenesis [114]. Hedgehog signaling is initiated by the binding of the Hh ligands Sonic hedgehog (Shh), Indian hedgehog (Ihh), or Desert hedgehog (Dhh) to their cell surface receptors Patched1 (PTCH1) or Patched2 (PTCH2). When activated, Hh receptors can be internalized. This conformation change in Hh receptors releases Smoothened (Smo) from their repression. Then, Smo can activate the Glioma-associated oncogene homologue (Gli) family of transcription factors: Gli1, Gli2, and Gli3. This step leads to the translocation of Glis into the nucleus to transduce Hh signaling (Figure 2) [115]. Multiple studies using zebrafish embryos have shown that Hh signaling plays an important role in cardiomyocytes’ proliferation during heart development. Thomas et al. showed that decreasing Hh signaling activity by genetic mutation in smo or the treatment of cyclopamine (CyA), a pharmacological inhibitor of Smo, creates a cardiomyocyte deficit; however, increasing Hh activity by the overexpression of shh or ptch1/ptch2 double knockout creates a cardiomyocyte surplus [116]. By using fluorescent ubiquitylation-based cell cycle indicator (FUCCI) technology, Choi et al. visualized cardiomyocyte proliferation events in live zebrafish embryos. The treatment of embryos with either Hh signaling Smo agonist (SAG) or antagonist (CyA) increased or decreased the number of proliferating cardiomyocytes, respectively [117]. In addition, a similar, conserved promoting role of Hh signaling has also been demonstrated in neonatal, adolescent, and adult mouse heart regeneration, and in the proliferation of hiPSC-derived cardiomyocytes [118].

### 3.6. Hippo Signaling

Hippo signaling, especially the Hippo–YAP axis, is a key regulator of organogenesis, governing cellular proliferation, differentiation, and apoptosis for organ size and growth. YAP and TAZ proteins function as co-activators to TEA domain transcription factor family members (TEADs), as well as various other related transcription factors like hypoxia inducible factors 1a (HIF1α) and 1B (HIF1β), octamer-binding transcription factor 4 (OCT4), Kruppel-like factor 5 (Klf5), Smad, TBX5, and β-catenin [119,120,121]. This enables the modulation of various proliferation and survival genes [119]. After an early proliferative period, Hippo signaling arrests YAP/TAZ in response to diverse, largely extracellular signals including mechanical tension, ECM homeostasis, cellular junctions, and G-protein coupled receptor activity. Once signals are received, key Hippo signaling mediators MST1/2 and their partner SAV1 are phosphorylated and act as kinases for downstream LATS1/2 and its adaptor MOB1. The activated LATS1/2 and MOB1 complex inactivates YAP/TAZ through phosphorylation (Figure 2). Initially studied in *drosophila*, MST1/2 and LATS1/2 mutations were shown cause excessive tissue growth [120,121]. Posttranslational modifications in response to specific stimuli also play an important role in modulating the activity of LATS1/2 and YAP/TAZ [121].

In the heart, YAP signaling contributes to the initial embryonic emergence of cardiomyocytes, while related Hippo signaling inhibits YAP and influences cardiomyocyte maturity and homeostasis in adulthood [119,121]. Hippo signaling is also essential for epicardium development, with LATS1/2 epicardial deletion being associated with coronary artery defects and embryonic lethality. YAP/TAZ epicardial deletion is also associated with coronary artery defects, demonstrating the importance of both ends of the Hippo–YAP signaling axis in cardiac development [119,121]. The Hippo pathway holds particular interest in cardiovascular medicine, as the increased activation of Hippo signaling later in life critically limits cardiomyocyte regeneration. Thus, Hippo signaling modulation, specifically its inhibition, has shown therapeutic promise for regenerating adult cardiomyocytes in the case of myocardial infarction and/or heart failure (Figure 2) [119,120]. In response to ischemia reperfusion injury following myocardial infarction, MST1 activation increases, leading to more apoptosis and potentially worsening the injury [121]. Researchers have found that this proliferation arrest in cardiomyocytes can indeed be reversed by simultaneous YAP and β-catenin activation. Even small-molecule drugs like TT-10 (C11H10FN3OS2) are being developed to reinitiate cardiomyocyte proliferation [121]. Using adeno-associated virus serotype 9 (AAV9) to activate YAP and, in another study, to inhibit SAV1 both improve recovery from cardiac injury [119].

### 3.7. Notch Signaling 

Heart development is a complex process that simultaneously relies on the contribution and crosstalk of various signaling pathways. The Notch signaling pathway is a highly conserved intercellular signaling pathway that plays a pivotal role in many developmental processes, including heart development. Notch signaling in the developing heart regulates cardiac specification, progenitor cell differentiation, valve primordium formation, ventricular trabeculation, ventricle wall compaction, and coronary vessel development [122]. In humans, Notch signaling dysregulation causes congenital heart defects or other developmental syndromes which affect the heart such as aortic valve calcification and Alagille syndrome [123,124,125,126]. More importantly, Notch signaling coordinates cellular interactions during heart development via crosstalk with key pathways, including the Wnt and TGF-β (BMP) signaling pathways. As described above, canonical Wnt and BMP signaling are required for early mesoderm formation. Later in development, canonical Wnt signaling inhibition is necessary for the cardiac specification of mesodermal progenitors. Notch signaling is required for the cardiac differentiation of progenitors by BMP signaling activation and the inhibition of canonical Wnt signaling [127]. Specifically, the Notch4 isoform’s expression is restricted to the endothelial component of the developing heart, promoting heart development through activation of the BMP signaling components BMP2, BMP6, and BMP7. The concurrent inhibition of canonical Wnt signaling was also required. It was achieved by the Notch4-induced expression of Wnt inhibitors, sFRP1, and sFRP5.

### 3.8. Cell Populations in Heart Development

Mammalian cardiogenesis is a complex process regulated spatially and temporally by many types of cardiac progenitor cells. Identifying distinct cardiac progenitor cell populations and elucidating how these cell populations are generated and interact with each other holds the key to better understanding the cardiac morphogenetic process. Model organisms have so far played an indispensable role in studying these cardiac cell populations. While mice are among the most common today, our modern understandings of the FHF and SHF are rooted in discoveries from chick embryos, a so-called “classic” model organism [128,129]. This model has historically played a key role in investigating cardiac development mechanisms, including the heart fields and later developmental anatomy like the neural crest and proepicardial organ. The macroscopic, ex utero nature of the chick embryo allows relatively easy observation of and intervention in organogenic events, a key benefit to researchers, and is a factor that made the model historically accessible [128,129]. This has contributed to knowledge not just about heart development, but the simultaneous development and cooperation of other organs [129]. For instance, cardiac neural crest cell contributions to the formation of the aortic-pulmonary septum and outflow tract, as well as the migration of the proepicardial organ from the foregut to heart and its contribution to linear heart tube folding. The chick embryo model was foundational in understanding the direct role of the proepicardial organ in producing coronary vasculature and the associated EMT-inducing factors VEGF, FGF, TGF-β, and PDGF-BB [128]. Since the establishment of the chick embryo model, our ability to produce specific mouse lines as well as grow human pluripotent stem cells in the lab has advanced our ability to investigate cardiac development. Nevertheless, the chick embryo still holds potential utility in the study of vertebrate biology [128]. 

Genetic lineage tracing in mouse development and embryonic stem cells has provided valuable insights into mammalian cardiogenesis. These studies revealed that Mesp-1, Isl1, and Nkx2.5 mark the cardiac progenitor cells that are indispensable for generating all major cell types in the mouse heart [22,25,130,131,132]. By analyzing histone modifications at promoters during the stepwise differentiation of mouse embryonic stem cells into CMs, Wamstad et al. characterized the dynamic epigenetic and transcriptional landscapes during cardiac differentiation [133]. Moreover, thousands of stage-specific enhancers that may define new transcriptional networks during cardiac differentiation were identified in the study by profiling H3K4me1 and H3K27ac histone markers. With the advancement of gene profiling technologies, especially next-generation deep RNA sequencing at the single-cell level (single-cell RNA-seq or ScRNA-seq), more and more lineage-specific cell populations have been identified and characterized [134,135]. To comprehensively assess the transcriptional profiles of single cardiac cells in the embryonic mouse heart, Li et al. developed Anatomical Transcription-based Legend from Analysis of Single-cell RNA-Sequencing (ATLAS-seq) [136]. This anatomically informed single-cell transcriptomic profiling study of 118, 949, and 1,166 cardiac cells from embryonic day 8.5 (E8.5), E9.5, and E10.5 of murine development revealed spatially patterned gene expression signatures in developing CMs. They segregated these cells by type using unsupervised bioinformatics analysis and identified chamber-specific genes. The spatial origins of single cardiac cells were reconstructed with very high accuracy by using a random forest algorithm. By analysis of the single-cell RNA sequencing of >1200 murine cells isolated at seven time points spanning from E9.5 (primordial heart tube) to postnatal day 21 (mature heart), DeLaughter et al. revealed the dynamic transcriptional programs which direct CM maturation during heart development. Using unbiased transcriptional data, the authors further classified CMs, endothelial cells, and fibroblast-enriched cells, and identified markers for temporal and chamber-specific developmental programs [137].

Given the evolutionary divergence between mice and humans [138], cardiac cell populations in the developing human heart may have different gene expression profiles compared to murine counterparts. Therefore, studies on human tissue are invaluable for insight into human heart development [139,140,141]. Asp et al. performed spatial transcriptomics (ST) at a single-cell resolution and in situ sequencing (ISS) in developing human heart samples at three developmental stages in the first trimester: 4.5–5, 6.5, and 9 weeks post-conception [139]. ST analysis in these samples identified unique gene profiles that correspond to distinct anatomical regions of the human heart in each developmental stage. ISS is a targeted approach that uses padlock probes and rolling circle amplification in tissue sections to target known genes. This enabled analysis with a subcellular resolution to confirm regional markers and cell type identifications based on ST and scRNA-seq. This study built the gene expression landscape of human heart development at the single-cell resolution and constructed a 3D organ-wide atlas. Similar approaches were applied to study the developing human heart at later stages (ranging from 5 weeks to 25 weeks post-conception) by Cui and colleagues [140]. This study not only systematically mapped the transcriptomic landscape of the human fetal heart at later stages, but also identified and characterized four major types of cells: CMs, cardiac fibroblasts, endothelial cells (ECs), and valvar interstitial cells (VICs). The single-cell transcriptomic data revealed several key, stand-alone pathways for heart development. Notch signaling was highly enriched in the endocardial cells of 7-week-old embryos to promote CM differentiation from the trabecular layer. The BMP signaling pathway was activated in ECs and fibroblasts during heart development from 5 weeks to 25 weeks to control their fates. Comparing human scRNA-seq data and mouse data collected from previous studies [136,142], significant differences in species-specific gene expression allowed researchers to identity human specific cardiac marker genes. For example, *RNASE1* was specifically expressed in ECs, *THY1* was highly expressed in fibroblasts, and *CFB* and *ITLN1* were predominantly expressed in epicardial cells (Eps), whereas these genes were barely expressed in the mouse heart. In contrast, *Icam2* was specifically expressed in ECs, and *Rnf213* was highly expressed in EPs in the mouse heart. Together, these studies provide valuable spatial and temporal gene profile maps for heart development and previously unidentified human-specific cardiogenic gene programming. 

## 4. The Development of Human Cell-Based System for Heart Diseases

Heart disease is a significant cause of death worldwide, with increasing prevalence. This urges the development of better models to study the pathogenesis of heart diseases and find new therapeutic strategies to battle these diseases. Discovering crucial signaling pathways which regulate heart development has enabled us to gain essential knowledge regarding heart formation and improve our heart disease models. 

Due to the ethical and legal limitations of research involving human subjects, animal models such as zebrafish and rodents are essential alternatives for studying human heart diseases. However, despite various advantages of using animal models to study human heart diseases, none of these animal models can perfectly recapitulate all aspects of complex human diseases. Significant differences exist between the human heart and those of model organisms, including the heart electrophysiology, cell size, multinucleation frequency, and myosin heavy chain expression [143,144]. The discordance between human and animal physiologies and pathophysiologies has contributed significantly to the approximately 89% failure rate of novel drugs in human clinical trials [145]. Human cell-based systems are considered more reliable in uncovering molecular mechanisms of human cardiac disorders and developing therapies to treat them. Therefore, creating a human cell-based system to study heart disease is urgent. The human cell system has other advantages beyond its human origin. First, the cell culture is highly scalable and has a higher throughput than using animal models. Second, the cultured cells are easily visualized and imaged using standard microscopes. Many cellular processes, such as cell adhesion, intracellular trafficking, and signal transduction, can be visualized at the molecular level with the help of advanced fluorescent labeling. These benefits are challenging to achieve in animal models [146]. Third, human cells can be derived directly from individual patients [147]. This is indispensable when studying unique types of heart conditions.

One of the biggest challenges in using human cell-based systems to model heart diseases is the limited source of primary cardiac cells. During the last two decades, advances in using human pluripotent stem cells (PSCs), including human embryonic stem cells (hESCs) and human induced pluripotent stem cells (hiPSCs), to generate cardiac cells has largely overcome this barrier [148,149].

### 4.1. Two Dimensional PSC-Derived Platforms

The mechanistic insights into heart development have significantly improved PSC to cardiac cell differentiation protocols. TGF-β and Wnt signaling are critical for mesoderm induction in gastrulation [150]. Adding TGF-βs to the culture medium during ES differentiation enhances cardiac differentiation [151,152]. The addition of a growth factor cocktail containing Activin, BMP4, and other growth factors such as basic fibroblast growth factor (bFGF) and vascular endothelial growth factor (VEGF) expanded the cardiac progenitor cell population differentiated from human ESCs [152]. Wnt signaling plays a biphasic role during cardiac differentiation. Canonical Wnt signaling activation is required for establishing cardiac progenitor populations in the early stage but suppresses cardiac differentiation and maturation in the later stage [54]. Lian et al. developed a robust cardiac differentiation protocol by modulating canonical Wnt signaling at different induction stages [153]. This protocol adds the GSK-3 inhibitor CHIR99021 to activate canonical Wnt/β-catenin signaling at the start of the differentiation of PSCs into mesodermal progenitors. In the later stage, a Wnt inhibitor, IWP2, is utilized to inhibit canonical Wnt/β-catenin signaling. By modulating Wnt signaling, 80–98% of the cells can be made to differentiate into functional CMs without cell sorting or selection. RA signaling plays essential roles in multiple stages of heart development, including restricting the cardiac progenitor pool, regulating anterior-posterior polarization of the heart, and CM subtype specification. Zhang et al. added RA or an RA inhibitor to cultures during the differentiation of human ESCs at different times. They investigated these factors’ effects on the cardiac subtype specification [109]. Results from this study show that RA signaling drives the differentiation of hESCs into atrial-like CMs, whereas RA signaling inhibition results in a relatively homogeneous population of ventricular-like CMs. 

Multiple studies have taken advantage of these efficient CM induction protocols for their heart disease modeling [154,155]. Left ventricular non-compaction (LVNC) is the third most prevalent cardiomyopathy in children and is caused mainly by defective embryonic myocardium development. hiPSC-CMs derived from a LVNC patient carrying a mutation in the *TBX20* gene, a candidate of genetic causes of LVNC, were used to model LVNC in vitro. Further investigation revealed that LVNC iPSC-CMs have a decreased proliferative capacity due to abnormal TGF-β signaling activation, due in turn to a defective TBX20–PRDM16 axis [154]. Lee et al. modelled LMNA-related dilated cardiomyopathy (DCM) in vitro using patient-specific iPSC-CMs. LMNA mutant iPSC-CMs displayed aberrant calcium homeostasis that led to arrhythmias, commonly seen in human LMNA deficient patients. Further mechanistic investigation showed that platelet-derived growth factor (PDGF) pathway activation contributes to LMNA-related DCM pathogenesis [155]. Danon disease can cause severe cardiomyopathy and heart failure through poorly defined mechanisms. Chi et al. modeled Danon cardiomyopathy in a dish with patient-derived hiPSC-CMs. Danon hiPSC-CMs recapitulated patient defects including decreased autophagic flux, mitochondrial, and contractile abnormalities, which allowed researchers to study the pathogenesis of this disease on a molecular level. This study revealed a causative role of mutations in the B isoform of the *LAMP-2* gene in Danon cardiomyopathy due to the failure in the autophagic fusion step [156]. Using this well-defined cardiac differentiation protocol, Chi et al. identified the downregulation of canonical Wnt signaling downstream of an increased dosage of interferon (IFN) receptor (IFNR) genes on chromosome 21 as a causative factor of cardiogenic dysregulation in Down syndrome (DS). Furthermore, the genetic and pharmacological normalization of IFN signaling restored canonical WNT signaling and rescued defects in DS cardiogenesis in vitro and in vivo. These findings not only provide mechanistic insight into abnormal DS cardiogenesis, but also elucidate the development of future therapeutic strategies [49].

In addition to CMs, PSCs can also be differentiated into cardiac fibroblasts (CFs), vascular smooth muscle cells (VSMCs), and endothelial cells (ECs). CFs mainly originate from epicardial cells (EPIs) [157]. A previous study has shown that cardiac mesoderm was induced from hiPSCs by activating both Wnt/β-catenin and TGF-β signaling. After day 3 of the induction, Wnt signaling was inhibited by XAV939, while RA signaling was activated alongside TGF-β/BMP4 signaling. On day 9, TGF-β inhibitor SB431542 was added to the medium to suppress TGF-β signaling. By day 12, EPIs with a typical epithelial, cobblestone-like morphology emerged. CFs were generated from EPIs by culturing them in medium supplemented with fibroblast growth factor 2 (FGF2) [158]. Activated cardiac fibroblasts are the major cell type that secretes excessive extracellular matrix, which contributes to pathological fibrosis and leads to heart failure. Multiple studies have used PSC-derived CFs to model cardiac fibrosis. Zhang et al. showed that human iPSC-derived cardiac fibroblasts (iPSC-CFs) closely resemble primary CFs at the transcriptional, cellular, and functional levels. This in vitro iPSC-CF platform can be used to explore the mechanisms of cardiac fibrosis. The authors found that these iPSC-CFs are sensitive to both pro- and anti-fibrotic drugs. Using iPSC-CF and iPSC-CM co-culturing, this study elucidates crosstalk between CMs and CFs through natriuretic peptides and their cognate receptor suppression of CF activation, thereby representing a new direction of anti-fibrotic therapy [159]. Duchenne muscular dystrophy (DMD) is a severe form of muscular dystrophy caused by X chromosome-linked mutations in the *dystrophin* gene [160]. Cardiac fibrosis is a critical pathological feature of the DMD heart [161]. Soussi et al. used DMD patient-derived iPSC-CFs to model cardiac fibrosis. The loss of full-length dystrophin, an actin cytoskeleton component, in DMD iPSC-CFs induced metabolic remodeling, causing increased fibroblast activation after pro-fibrotic stimulation. This study highlights the relationship between cytoskeletal dynamics, metabolism, and myofibroblast differentiation while providing a new mechanism by which the loss of dystrophin in CFs may increase the severity of cardiac fibrosis [162].

VSMCs are the predominant cell type residing in the medial layer of major blood vessels and play a critical role in maintaining vascular wall integrity and blood pressure. VSMCs are derived predominantly from mesoderm and secondarily from neuroectoderm (neural crest, NC) cell lineages [163,164,165]. PSCs can be differentiated into VSMCs through the non-specific embryoid body (EB) cell aggregation method followed by culturing EBs in SMC-specific medium [166]. However, VSMCs derived from PSCs in this protocol were usually of an unknown lineage origin and had limited VSMC markers. Lineage-specific differentiation can be achieved by way of mesodermal or neuroectoderm intermediates, leading to the induction of neural crest (NC), lateral plate mesoderm (LPM), or paraxial mesoderm (PM) cell populations [167]. NC differentiation could be initiated in PSC monolayers using chemically defined medium (CDM) supplemented with polyvinyl alcohol (PVA), FGF, and SB431542 (an ALK5 TGF-β/Activin/Nodal pathway inhibitor); early mesoderm differentiation could be initiated by CDM-PVA medium with FGF-2, LY294002 (an inhibitor of phosphoinositide 3-kinases (PI3K)), and BMP4. LPM could then be induced by supplementing CDM-PVA medium with FGF2 and BMP4; or PM could also be induced by CDM-PVA medium with FGF2 and LY294002. After specific cell lineage induction, lineage-specific VSMCs could be generated by supplementing precursors with platelet-derived growth factor (PDGF)-BB and TGF-β1 in CDM, followed by culturing in SMC-specific medium. VSMCs differentiated from all these lineages showed characteristic spindle-shaped VSMC morphologies and expressed VSMC markers including Transgelin (TAGLN), Calponin 1 (CNN1), and smooth muscle myosin heavy chain (MYH11). PSC-derived VSMCs in experimental biology have advanced cardiovascular pathogenesis modeling. Modeled diseases include supravalvular aortic stenosis (SVAS) accompanied with narrowing or blockage of the ascending aorta and other arterial vessels caused by mutations in elastin (ELN) [168]; congenital cardiovascular malformation associated with bicuspid aortic valve (BAV) defects [169]; Marfan’s syndrome (MFS) due to the development of thoracic aortic aneurysm (TAA) [170]; and cardiovascular calcification caused by transformed VSMCs which secrete mineralizing extracellular vesicles that form microcalcifications [171]. Interestingly, Gao et al. recently tested the angiogenic potential of hiPSC-derived VSMCs [172]. hiPSC-VSMCs were injected intramuscularly into a murine hindlimb ischemia model. Functional outcomes and the blood flow were improved in hiPSC-VSMC-treated mice compared with controls. hiPSC-VSMC-treated mice showed an increased expression of VEGF in ischemic limbs, which leads to elevated VEGF-mediated angiogenesis. 

ECs line the inner walls of all blood vessels in contact with blood and play a critical role in the regulation of vascular permeability, angiogenesis, and tissue regeneration [173]. ECs originate from the mesoderm [174]. Like VSMCs, ECs can be generated, albeit at a very low yield, from either spontaneously differentiating EBs or treating EBs with a combination of growth factors including Activin A, BMP4, bFGF, VEGF, and dickkopf homolog 1 (DKK1) [152,175]. Recent studies have developed more efficient protocols to differentiate ECs from mono-layer PSCs. Lian et al. showed that treating hiPSCs with Wnt signaling activator CHIR99021 alone, in the absence of cytokines, can generate mesodermal cells. Subsequentially culturing these progenitor cells in endothelial growth media (StemPro-34 medium supplemented with VEGF) generates ECs [176]. Hamad et al. then further modified this method by treating mesodermal progenitor cells with a combination of human VEGF, bFGF, 8-Bromoadenosine 3′,5′-cyclic monophosphate sodium salt monohydrate (8Bro), and melatonin [177]. This method generates PSC-derived ECs with over a 90% purity in as little as 6 days. Taking advantage of these highly efficient differentiation approaches, patient-derived hiPSC-ECs have been widely applied to cardiovascular disease modeling [178]. For example, mutations in BMPR2 (bone morphogenetic protein receptor type II) can cause pulmonary arterial hypertension (PAH). BMPR2-deficient patient-derived hiPSC-ECs exhibited decreased tube formation, LDL uptake, adhesion, migration, and survival. Increasing specific BMPR2 activators, reducing BMPR2 inhibitors, or correcting BMPR2 mutations with genome editing can rescue the defects observed in patient-derived hiPSC-ECs [179]. Additionally, hiPSC-ECs have been used to evaluate the cardiotoxicity of chemotherapeutic compounds. Sharma et al., through a high-throughput screening, identified that vascular endothelial growth factor receptor 2 (VEGFR2)/platelet-derived growth factor receptor (PDGFR)-inhibiting tyrosine kinase inhibitors caused cardiotoxicity in hiPSC-ECs [180]. 

### 4.2. Cardiac Organoids Derived from PSCs

Animal models of heart disease can be used to examine cardiac pathogenesis in intact, multi-cell type, physiological environments, which is not feasible for in vitro cell-based systems. The heart contains various cell types, including CMs, endothelial cells, smooth muscle cells, fibroblasts, epicardial cells, and endocardial cells [148]. Communication between cell types is crucial for maintaining heart function [181]. Therefore, integrating different cardiac cell types in three-dimensional (3D) culture can better reflect the complexity of the in vivo heart tissue. Culturing in 3D has the benefit of replicating more life-like signaling between cells. Take for instance the case of EVs, which are released at much higher volumes in 3D compared to 2D cultures [31]. It has been noted that the secretion of EVs to facilitate crosstalk between cell types is mechanistically significant, and its further investigation may enable more fine-tuned control over cardiac organoids and spheroids [31]. EVs obtained from patients’ healthy, diseased, or otherwise stimuli-conditioned tissue may even be used to establish particular phenotypes in cardiovascular models or patients [27,31,32,182,183]. Giacomelli et al. induced the cardiac mesoderm in a monolayer culture by activating Wnt/β-catenin and TGF-β signaling using CHIR99021, Activin, and BMP4. Then, CMs and endothelial cells were generated by adding Wnt signaling inhibitor XAV939 and VEGF. These two cell populations were enriched and mixed, producing beating 3D structures termed cardiac microtissues (CMTs) [184]. Recently, the same group added cardiac fibroblasts (CFs) to their CMTs [158]. The authors demonstrated that CMT structural, electrical, mechanical, and metabolic maturation properties were further enhanced by including CFs. Interestingly, including CFs derived from arrhythmogenic cardiomyopathy patient-specific iPSCs in healthy CMTs recapitulated the patient’s arrhythmias, indicating that non-CMs affect the behaviors of CMs. These findings suggest that 3D structures combining various types of cardiac cells are promising in vitro models for human heart disease. 

**Figure 3:**
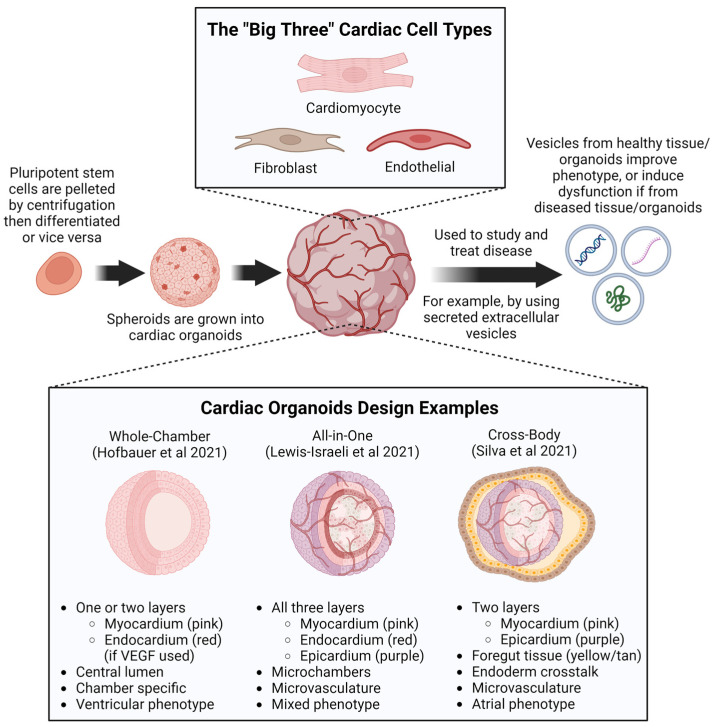
Recent cardiac organoid advances: Cardiac organoids are a type of constructed cardiac tissue most often derived from PSCs. To produce cardiac organoids, PSCs are pelleted by centrifugation and are then cultured in factor-specific media to accomplish specific cell-type differentiation. Sometimes this process is reversed, with PSC monolayers being differentiated into desired cell types, then mixed and pelleted as a composite. Cardiac organoids seek to replicate important aspects of heart physiology, particularly producing the “big three” cardiac cell types: cardiomyocytes, fibroblasts, and endothelial cells. Once an organoid is established, they can be used as a versatile platform to study disease in and test treatments on. Notable cardiac organoid designs have been established over the past few years. Among these are whole-chamber [185], all-in-one [186], and cross-body [187] cardiac organoids which recapitulate various layers of the heart, chamber-like morphology, and often microvasculature and a diversity of cell types. Of particular note are extracellular vesicles (EVs), which are increasingly recognized as an essential mediator of cellular communication and crosstalk. EVs harvested from physiological/healthy organoids tend to improve the health of treated tissue. Conversely, EVs obtained from pathological/diseased organoids tend to worsen the health of treated tissue. EV, extracellular vesicle; PSC, pluripotent stem cell; VEGF, vascular endothelial growth factor.

While cardiac organoids and spheroids may be manipulated by the introduction of specific EVs, EVs may also be harvested from patients or cardiac tissues to be used as potential therapeutics [27,182,183]. Recently, a combination of studies investigating cardiac EVs from healthy patients versus those from patients exposed to remote/non-cardiac ischemia were performed. EVs from healthy hearts were able to improve the viability in hypoxia-treated cells and ischemia-reperfusion injury recovery in rat hearts in part, at least, through STAT3 activation [182,183]. This suggests that EVs support healthy tissue homeostasis and repair by delivering several bioactive factors (growth factors, other proteins, sugars, lipids, mRNA, miRNA, DNA, etc.) which help to replicate the conditions under which the EVs were produced [31,32,183]. That is to say, EVs from healthy tissue appear to be beneficial signaling mediators, and EVs from diseased tissue appear to be detrimental signal mediators (Figure 3). This can be seen when stem cell-derived EVs reverse phenotypic signs of aging through antioxidant delivery [31]. A supporting example in the opposite direction comes from a mouse study which demonstrates that maternal injection with exosomes from diabetic mice increases the risk of CHD among offspring. Similarly, tumor-derived EVs promote a premetastatic niche [31]. In fact, exosomes, a type of EV, have been loaded into cardiac patches in recent clinical trials, and have even been harvested from in vitro cardiac tissues including cardiac organoids and spheroids [27,32]. Life-like cardiac organoids and spheroids provide a well-suited platform to study both the production and impacts of EVs on signaling pathways. This is evidenced in the fact that 3D tissues, compared to 2D tissues, produce around three times more exosomes and a shift towards a lower protein content and higher miRNA content of EV cargo [31].

While spheroids and organoids possess several applications, those generated by the co-differentiation or co-culturing of cells rely on the self-aggregation of differentiated cells, often lacking the structural complexity of the human heart. Seeding iPSCs onto a geometrically confined micropatterned surface, Ma et al. introduced spatial, biophysical cues, generating cardiac organoids with chamber-like structures (microchambers) [188]. In these organoids, cells in the center differentiated into CMs while cells on the perimeter differentiated into myofibroblasts. Interestingly, small cell-free regions have been observed inside these organoids. These cardiac microchambers, reminiscent of early heart development, could be used to model early human heart development in vitro. Recently, Hofbauer et al. found that adding ECM proteins (laminins 521/511) involved in mesoderm development to the media before mesoderm induction resulted in the intrinsic self-assembly of cells into beating 3D microchambers from 2D culture [185]. These microchambered organoids feature key hallmarks of self-organization: continuous specification, intrinsic mesoderm patterning, and self-directed morphogenesis into heart architecture, with microchambers analogous to the heart tube and early left ventricle (Figure 3). After inflicting cryoinjury to model developmental injury, organoids displayed increased fibronectin and collagen accumulation, which is consistent with in vivo pathophysiology. Thus, these organoids represent a powerful platform to mechanistically dissect self-organization, congenital heart defects, and response to injury. Recently, Lewis-Israeli et al. developed a relatively low-cost method to generate human heart organoids [186]. These organoids were generated from embryoid bodies (EBs) that were subject to three sequential Wnt signaling modulation steps (activation/inhibition/activation). Wnt signaling was activated at the early stage of differentiation (day 0 to day 2) then inactivated to allow the formation of cardiac mesoderm. A second Wnt activation step for 1 h on differentiation day 7 increased the organoid complexity, producing more developmentally relevant structures like the epicardium and microvessels. Heart organoids generated by this method not only displayed heart-like structures in terms of structure, organization, cardiac cell type complexity, ECM composition, and vascularization, but are also comparable to age-matched human fetal cardiac tissues at the transcriptomic, structural, and cellular level (Figure 3). Interestingly, as proof-of-concept of the platform’s utility, these organoids were used to model the effects of pregestational diabetes (PGD) on heart development. First-trimester maternal diabetes has been linked to a high CHD occurrence in newborns [189]. To model PGD, the levels of glucose and insulin in the culture medium were manipulated to reflect physiological levels in normal mothers and females with type I and type II pregestational diabetes. Normal heart organoids and PGD heart organoids showed significant morphological differences as early as day 4 of differentiation. Additionally, consistent with the observations in patients, PGD culture increased oxidative stress, cardiomyopathy, and altered lipid profiles. These findings suggest that this heart organoid platform is a useful platform for studying CHDs and their potential pharmacological treatments. Recent advances in cardiac organoids are illustrated in Figure 3.

## 5. Challenges and Perspectives

The heart is a complicated organ composed of many types of cells. Cardiac cells are equipped with specific receptors and complex intracellular complexes, allowing the heart to respond to extracellular stimuli precisely. The unique four-chamber structure requires the spatio-temporal coordination of many cellular processes regulated by signaling pathways. At the whole-body level, the heart receives signals from other organs/systems, such as the brain [190], immune system [191,192], kidney [193], liver [194], and adipose tissues [195]. Many pathological conditions, including cancer [196], diabetes [197], and neurodegenerative diseases [198], affect heart health. Therefore, to better understand the pathogenesis of heart disease, much effort is needed to investigate signal transduction within the cardiovascular system and within the whole body. To model the crosstalk between cardiac and non-cardiac cells, Silva et al. recently developed an in vitro system by generating organoids containing cardiac and gut cells from human iPSCs [187]. In contrast to the conventional induction protocol, which mainly produces cardiac cells, adding ascorbic acid and removing insulin in the culture media after differentiation day 7 promotes the formation of multilineage spheroids with a core of vigorously contracting cells surrounded by non-contracting cells (Figure 3). During prolonged culturing up to 100 days, these organoids undergo parallel human embryonic heart and gut development, including the initial emergence of myocardial tissue followed by epicardial and gut tissue, with interstitial tissue in between. The co-emergence of gut tissue supports the physiologic maturation of human atrial/nodal CMs. This organoid model could be suitable for studying the paracrine input of surrounding tissues to the heart tissue.

CMT and organoid systems have significant advantages compared to the 2D system. However, the heart is not the shape of a spheroid. Well-developed chambers are required for function. Therefore, developing an organoid with heart architecture faces challenges. With the advances in bioengineering technologies, especially 3D bioprinting, many strategies for building 3D heart-like organoids have been developed. Generating cardiac organoids with carefully controlled biocompatible scaffolds such as patches, strips, rings, engineered heart tissues (EHTs) with micro-molds, and chambers has been achieved [185,199,200]. These efforts bring new possibilities for generating heart-like structures in a dish closer to reality. However, generating most of these organoids requires special and dedicated instruments. Making these organoids on a large scale faces challenges as well. Additionally, the nutrient availability throughout the organoids is uneven, frequently resulting in a necrotic core and limiting the maximum possible size of the organoid, which may be overcome by the application of microfluidic devices. Improving the vascularization of organoids also remains a significant challenge.
